# Identifying Lanthionine Ketimine Derivatives for Maturation and Proliferative Effects in Oligodendrocyte Progenitor Cells

**DOI:** 10.1080/17590914.2025.2535963

**Published:** 2025-07-21

**Authors:** Zachary McDonald, Ankit Tandon, Travis T. Denton, Mehek Taneja, Jacqueline Rocha, Jeffrey L. Dupree, Pablo M. Paez, Veronica T. Cheli, Swathi G. Tumuluri, Douglas L. Feinstein

**Affiliations:** a Department Anesthesiology, University of Illinois, Chicago, Illinois, USA; b Department of Pharmaceutical Sciences, College of Pharmacy & Pharmaceutical Sciences, Washington State University Health Sciences Spokane, Spokane, Washington, USA; c Department of Translational Medicine and Physiology, Elson S. Floyd College of Medicine, Washington State University Health Sciences Spokane, Spokane, Washington, USA; d Steve Gleason Institute for Neuroscience, Washington State University Health Sciences Spokane, Spokane, Washington, USA; e Department of Anatomy and Neurobiology, Virginia Commonwealth University, Richmond, Virginia, USA; f Research Service, HH McGuire VA Medical Center, Richmond, Virginia, USA; g Institute for Myelin and Glia Exploration, Department of Pharmacology and Toxicology, University at Buffalo, Buffalo, New York, USA; h Research Service, Jesse Brown VA Medical Center, Chicago, Illinois, USA

**Keywords:** differentiation, myelin, oligodendrocyte, Oli-neu cells, proliferation

## Abstract

Previous studies have shown that lanthionine ketimine ethyl ester (LKE) reduces clinical scores in the experimental autoimmune encephalomyelitis (EAE) mouse model of Multiple Sclerosis, induces differentiation of oligodendrocyte progenitor cells (OPCs) in vitro, and accelerates remyelination following cuprizone induced demyelination. In a search for derivatives with greater efficacy to induce OPC maturation or proliferation, we screened a panel of 2-alkyl and 3-phosphonate substituted LK derivatives. Incubation of Oli-neu oligodendrocyte cells with 2-*n*-butyl- or 2-*n*-hexyl-LKE-phosphonate reduced spontaneous cell death, increased proliferation, and increased maturation. These were associated with changes in corresponding mRNA levels of Olig2, PLP, and O4. These derivatives also reduced cell death and increased proliferation and maturation in primary mouse OPCs. The increased hydrophobicity of these derivatives suggests these will be better candidates for testing effects in animal models of Multiple Sclerosis and other demyelinating diseases.

## Introduction

Lanthionine is a non-proteogenic amino acid synthesized via transulfuration of cysteine with serine or a 2nd cysteine by cystathionine-b-synthase. Lanthionine is a substrate for glutamine transaminase K which yields an intermediate that cyclizes to form lanthionine ketimine (LK). LK can be derivatized to yield the ethyl ester LKE which has increased cellular permeability. Previous studies showed that LKE promotes neurite elongation, protect neurons against oxidative stress; suppresses microglial activation; and protects motor neurons from microglial-induced toxicity (Hensley et al., 2013; Nada et al., 2012). LKE also provides beneficial effects in mouse models of neurodegenerative diseases and conditions including AD (Hensley et al., 2013; Koehler et al., 2018), ischemia (Nada et al., 2012), ALS (Khanna et al., 2012), and spinal cord injury (Kotaka et al., 2017), and Parkinson’s disease (Togashi et al., 2020).

Proteomic studies (Hensley et al., 2010b) showed that LKE binds to synaptosomal membranes, and HPLC-MS/MS analysis identified a primary target as CRMP2 (Collapsin Response Mediator Protein 2). CRMP2 has been well characterized with respect to stimulation of neuritogenesis and axonal guidance (Kotaka et al., 2017; Quach et al., 2015; Zhang & Koch, 2017), and has roles in optic nerve damage in EAE (Mimura et al., 2006; Petratos et al., 2012). CRMP2 is an adaptor protein that interact with various binding partners including tubulin, and is involved in regulation of neurite growth and retraction; differentiation; and axonal transport. CRMP2 functions are regulated by phosphorylation, including at Serine 522 (S522) by Cdk5, cyclin dependent kinase-5 (Uchida et al., 2005). LKE increases CRMP2 functions (Hensley et al., 2010a; 2010b) by reducing Cdk5-mediated phosphorylation at S522 (Wilson et al., 2014). While primarily expressed in neurons in adult brain, CRMP2 is also expressed in OLGs, and roles in OLG survival, maturation and process extension have been described (Piaton et al., 2011; Syed et al., 2017).

LKE has also been shown to provide benefit is several mouse models of MS (Dupree et al., 2015; 2022). In the experimental autoimmune encephalomyelitis (EAE) mouse model of MS, we showed that treatment with LKE (initiated at the peak of disease) reduced clinical severity and neuroinflammation, and in the optic nerve and spinal cord reduced neurodegeneration and increased myelin thickness (Dupree et al., 2015). In cell culture, we showed that LKE exerts neuroprotective and neuritogenic actions on primary neurons (Marangoni et al., 2018); and promoted maturation of oligodendrocyte (OLG) progenitor cells (OPCs) (Savchenko et al., 2019). Based on those results, we tested if LKE could influence remyelination following chemical induced demyelination with the copper chelator cuprizone (CPZ) (Dupree et al., 2022). While spontaneous remyelination occurs in this model after cessation of CPZ treatment, in the presence of LKE the extent of remyelination was greater, and the distribution of myelin thickness around axons in the corpus callosum (CC) was similar to that observed in control mice.

While promising, the potential use of LKE as a therapeutic option is challenged by its relatively short half-life, limited CNS penetration, and metabolic breakdown. To address these issues, a panel of LK derivatives were generated in attempt to identify ones with improved chemical stability, membrane permeability, and functional activity (Gonzalez Porras et al., 2023; Shen et al., 2018). A number of these derivatives exhibit increased hydrophobicity suggesting they will have increased membrane permeability (Shen et al., 2018). Functional testing of several of the 2-substituted LKEs and 3-phosphone LK(E) analogues revealed that several are up to 10-fold more potent than LKE in their ability to increase autophagy, a known biological action of LKE (Hensley et al., 2016). In the current study we screened a panel of LK derivatives in mouse Oli-neu cells, a commonly used surrogate to examine OPC maturation (Boccazzi et al., 2023; de Faria et al., 2019; Enders et al., 2023; Gregorio et al., 2024). Our findings suggest that two derivatives, 2-*n*-butyl-LKE(P) and 2-*n*-hexyl-LKE(P), show comparable or superior effects as compared to LKE. Similar effects on proliferation and maturation were observed in primary mouse OPCs. Together the data suggests that these derivatives may be better candidates to pursue for therapeutic interventions, based on their having increased hydrophobicity which would predicts greater membrane permeability and ability to cross the blood brain barrier.

## Materials and Methods

### Lanthionine Ketimine Derivatives

LK-phosphonates (LK-P), LK-ester (LKE), and LKE-phosphonates (LKE-P) were synthesized using standard Michaelis-Arbuzov reaction conditions as described. Analog structures were confirmed by ^1^H, ^13 ^C and ^31^ P NMR and liquid chromatography tandem UV spectrophotometry high-resolution mass spectrometry (LC/UV/HRMS) (Shen et al., 2018).

### Oli-Neu Cell Cultures

Oli-neu cells were maintained in DMEM high glucose supplemented with 1% heat inactivated horse serum (Thermo Fisher cat. # 26050070), 10 mg/mL BSA, 1x N1 supplement (Sigma-Aldrich cat. #N6530), and 1x antibiotic/antimycotic (Corning cat. #30004CI) on poly-D-lysine coated flasks. The media was changed every 2-4 days, and cells passaged every 5-7 days. For immunostaining the cells were plated onto poly-D-lysine coated 8-well chamber slides.

### Primary Cultures of OPCs

Primary cultures of cortical OPCs were prepared as described (Cheli et al., 2015). First, cerebral hemispheres from 1-day old mice were mechanically dissociated, then plated in poly-D-lysine-coated flasks in DMEM/F12 (1:1 v/v) (Invitrogen) supplemented with 10% fetal bovine serum (FBS) (Life Technologies). After 4h the medium was changed and the cells were grown in DMEM/F12 supplemented with insulin (5 μg/ml), apotransferrin (50 μg/ml), sodium selenite (30 nM), d-biotin (10 mM) and 10% FBS (Life Technologies). Every 3 days 2/3 of the culture media was changed. OPCs were purified from the mixed glial culture after 14 days by a differential shaking and adhesion procedure and allowed to grow on poly-D-lysine-coated coverslips in DMEM/F12 supplemented with insulin (5 μg/ml), apotransferrin (50 μg/ml), sodium selenite (30 nM), 0.1% BSA, progesterone (0.06 ng/ml) and putrescine (16 μg/ml) (Sigma). OPCs were kept in mitogens, PDGF and bFGF (20 ng/ml) (Peprotech), for 2 days and then induced to differentiate by mitogen withdrawal and addition of T3 (15 nM) alone or with 25 µM LKE or LKE analogs for 2 days.

### Immunocytochemical Staining

Cells in poly-D-lysine coated wells or slides were rinsed with Dulbecco’s phosphate buffered saline (DPBS) and fixed with 4% paraformaldehyde for 20 minutes. Following fixation, citrate buffer was added at 60 °C for 5 minutes. Cells were blocked in 2% BSA for 1 hour, before addition of primary antibodies (Table 1) for 2 hours. Antibodies were removed, cells washed 4 times in DPBS, followed by the addition of appropriate secondary antibodies (Table 1) for 1 hour. Cells were washed again in DPBS, counter-stained with DAPI for 10 minutes, then a coverslip added over mounting medium (Vectashield cat #H-1000). Cells and reagents were kept at 37 °C for the entire procedure.

**Table 1.** Antibodies used.

Target	Source	Catalog #	Host	Clonality	Dilution
PDGFRα	Abcam	ab203491	Rabbit	mAb	1:300
Ki67	Thermo Fisher	MA5-14520	Mouse	mAb	1:300
Olig2	Abcam	ab109186	Rabbit	mAb	1:300
O4	Novus	MAB1326	Mouse	mAb	1:300
PLP	Abcam	Ab254363	Rabbit	mAb	1:300
α-rabbit IgG	Jackson Labs	711-585-152	Donkey	pAb	1:300
α-mouse IgG	Jackson Labs	711-585-151	Donkey	pAb	1:300
α-rabbit IgG	Jackson Labs	711-545-152	Donkey	pAb	1:300
α-mouse IgG	Jackson Labs	711-545-140	Donkey	pAb	1:300

### Image Quantitation

Following immunostaining, images were captured on a Keyence BZ-X810 microscope (Keyence, Osaka, Japan), and the number of positively stained cells was determined using included software (Keyence Analyzer Hybrid Cell Count). For all studies, at least 10 fields per chamber of an 8-chamber slide were captured at 20x magnification. Background staining was subtracted; and the # of positive features in the whole field was calculated for each channel using included Keyence software.

### mRNA Quantitation

Cells were homogenized in RNAzol RT (Molecular Research Center, cat #RN190) and RNA was precipitated according to manufacturer’s protocol. One μg of total RNA was converted to cDNA using the High Capacity cDNA Reverse Transcription Kit (Applied Biosystems, cat #4368814). cDNA samples were amplified using primers specified in Table 2, using SsoAdvanced Universal SYBR Green Supermix (BioRad, cat #1725272) in a BioRad CFX96 real time PCR machine (BioRad, Hercules, CA). Ct values were exported and analyzed by the 2ΔΔCt method and normalized to β-actin.

**Table 2.** Primer sequences.

Target	Forward	Reverse
β-Actin	GCTTCTTTGCAGCTCCTTCGT	ATATCGTCATCCATGGCGAAC
MBP	CCTCCGTAGCCAAATCCTG	ACCCAAGATGAAAACCCAGTAG
PLP	TCAGCCGCAAAACAGACTAG	CACTCCAAAGAAACACAATCCAG
Olig2	GCGAGCACCTCAAATCTAATTC	AAAAGATCATCGGGTTCTGGG
PDGFRα	ACCCTCTATCCTCCCAAACGA	TCTCCCCAACGCATCTCAG
O4	CCCGGAGAAAATGAGATACTGATAG	ACACTGGAGAGGACAAATGC

### Cell Death Assay

Cell death was determined as the ratio of lactate dehydrogenase (LDH) in the supernatant to total LDH measured after cells were lysed (CytoTox 96^®^, Promega) according to manufacturer’s instructions.

### Data Analysis and Statistical Comparisons

The percentage of positively stained cells for Ki67, PDGFRα, O4, and Olig2 relative to the total number of cells as detected by DAPI was determined for all wells and chambers having at least 10 cells. The % staining was adjusted to 100% for any samples where DAPI staining was masked by overlying staining for other antigens. Analysis of cell death was done by two-way repeated measures ANOVA and Dunnett’s multiple comparison post hoc tests comparing analogs to control cells. Immunostaining results were compared by 1-way ANOVA and post hoc comparisons made using Fisher Dunnett’s multiple comparison post hoc tests. Graphpad Prism version 10.5 was used for statistical analyses.

## Results

**Figure 1.**
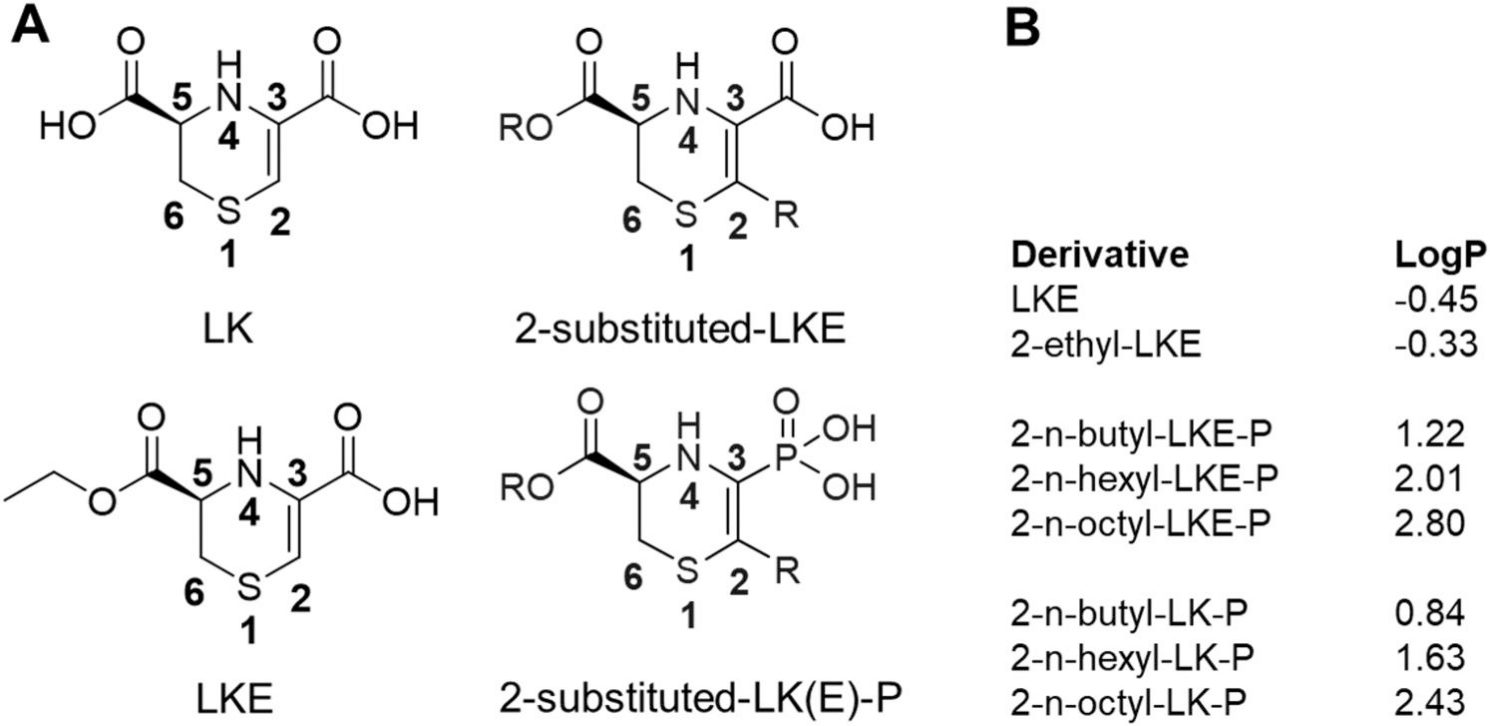
Chemical Structures of Lanthionine Ketimine analogues. **(A**) Structures of LK, LKE, and phosphonate derivatives with possible 2-substitutents. (**B**) LK analogues used in this study with calculated LogP values

The LK molecule can be modified in various ways including by addition of an ethyl group on the 5-ester; addition of alkyl groups at the 2-position; and addition of a phosphonate group at the 3-position (Figure 1A). Substitution with longer alkyl chains leads to a corresponding increase in hydrophobicity (LogP) which could increase the derivative’s ability to enter cells through membrane diffusion. We synthesized 8 derivatives (Figure 1B) to screen for relative efficacy to induce proliferation or maturation in the Oli-neu cell line and in primary mouse oligodendrocyte progenitor cells (OPCs).

**Figure 2.**
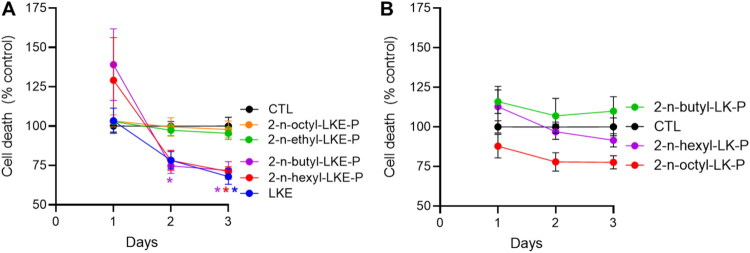
Relative toxicity of LK derivatives in Oli-neu cells. Oli-neu cells were incubated with 10 µM of (**A**) LKE and LKE-P derivatives and (**B**) LK-P derivatives, then cell death measured after indicated days by LDH release assay. For each day data is presented as the % cell death compared to untreated (CTL) cells, which was 16%, 18%, 14% on days 1, 2, and 3 respectively. Data is mean ± se of n = 5 per group. On day 2 cell death was reduced by 2-*n*-butyl LKE-P (BUT); and on day 3 by LKE, BUT, and 2-*n*-hexyl LKE-P (HEX). *, p < 0.05 versus CTL, 2-way repeated measures ANOVA with Dunnett’s multiple comparison test.

In Oli-neu cells, over the course of 3 days there was a basal degree of cell death amounting to between 14% and 18% (Figure 2). After 1 day, there were non-significant increases in relative cell death in the presence of 2-*n*-butyl-LKE-P (BUT) and 2-*n*-octyl-LKE-P (Figure 2A). However, after 2 days BUT significantly decreased cell death, and there was reduced cell death in the presence of LKE or 2-*n*-hexyl-LKE-P (HEX). After 3 days cell death was significantly reduced by LKE, BUT, and HEX. There were no significant effects on cell death versus untreated (CTL) cells in the presence of any of the LK-P derivatives tested (Figure 2B).

**Figure 3.**
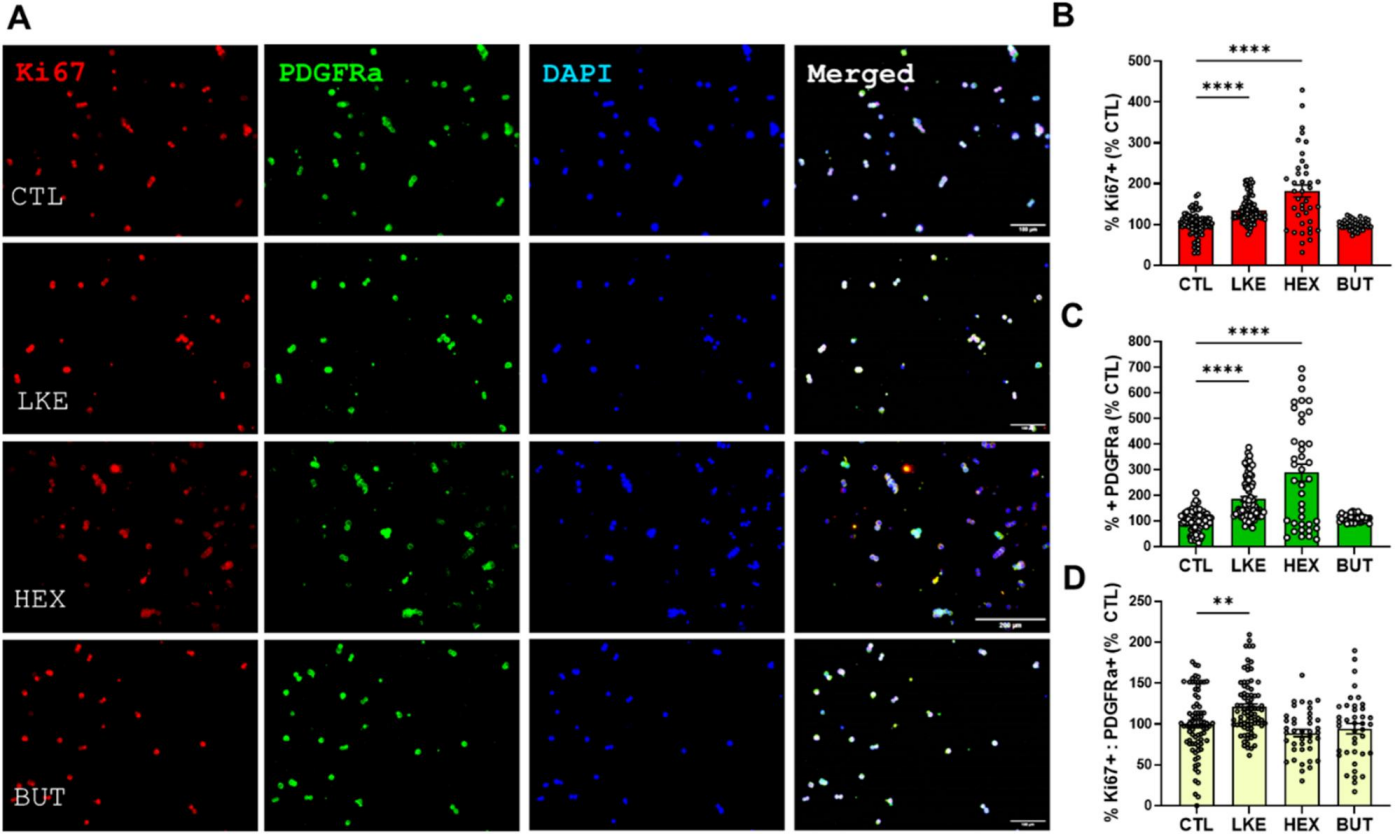
Incubation with LKE or HEX increases Oli-neu cell proliferation. (**A**) Representative images of Oli-neu cells after incubation with 10 µM LKE, HEX, BUT, or nothing (CTL) for 72 hours, then stained for Ki67 (red) or PDGFRα (green), and counterstained with DAPI (blue). Incubation with LKE or HEX increased both (**B**) Ki67 and (**C**) PDGRFα staining versus CTL, whereas only LKE (**D**) increased the % of PDGFRα+:Ki67+ double labeled cells. Data is mean ± se percent staining derived from 10 fields of view from each of 8 wells (CTL, LKE) or 4 wells (HEX, BUT), and normalized to staining in CTL cells in which of the total (DAPI stained) cells 52% were Ki67+, 43% were PDGFRα+, and 50% were doubled-labeled cells for Ki67 and PDGFRα. ****, p < 0.0001 versus CTL, 1-way ANOVA with Dunnett’s multiple comparison test.

**Figure 4.**
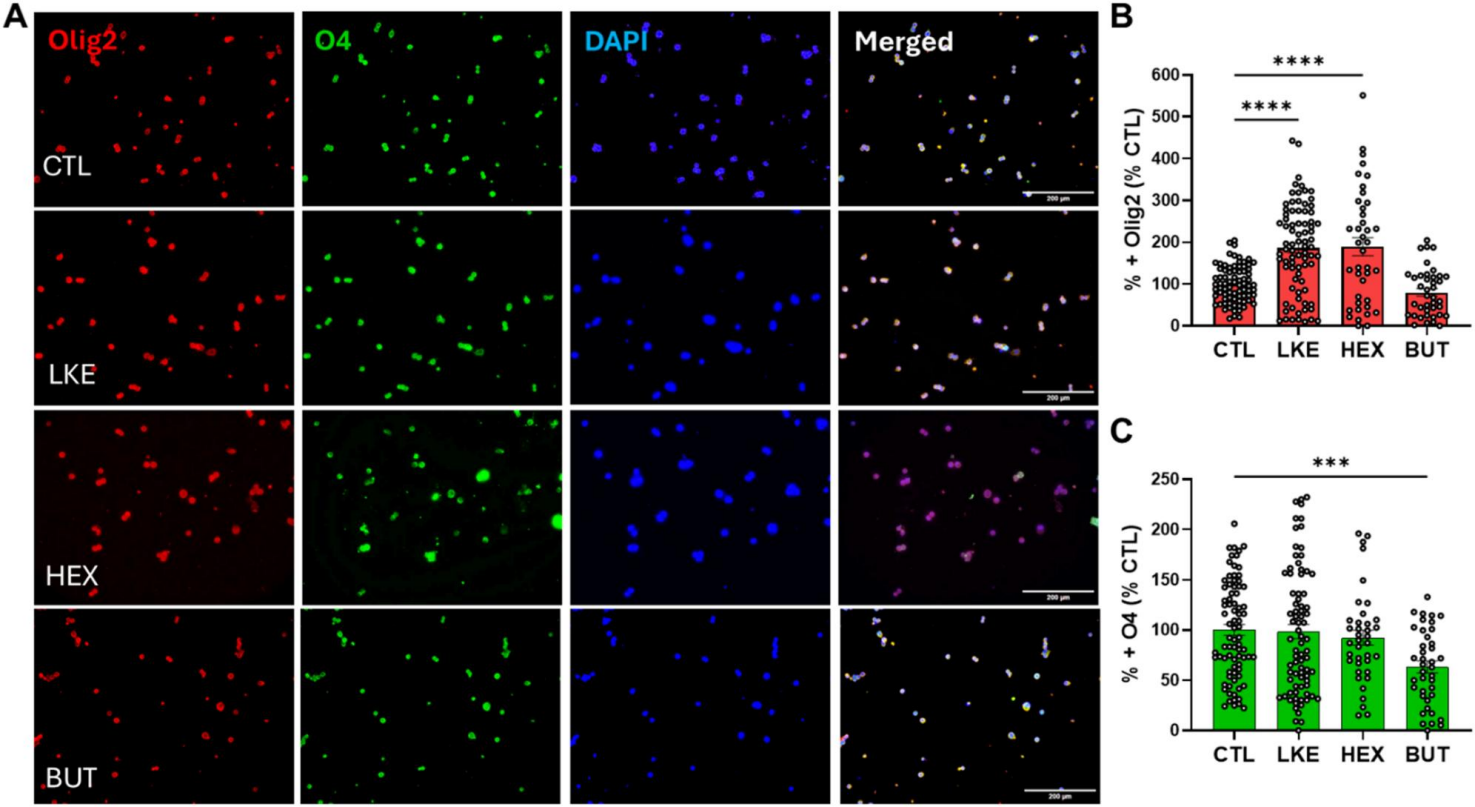
Incubation with LKE or HEX increases total oligodendrocyte numbers. (**A**) Representative images of Oli-neu cells following incubation with 10 µM LKE, HEX, BUT, or nothing (CTL) for 72 hours, then stained for Olig2 (red) or O4 (green), and counterstained with DAPI (blue). (**B)** Incubation with LKE or HEX increased Olig2 staining. (**C**) Incubation with BUT decreased O4 staining. Data is mean ± se percent staining derived from 10 fields of view from each of 8 wells (CTL, LKE) or 4 wells (HEX, BUT), and normalized to staining in the CTL cells in which of the total (DAPI stained) cells 33% were O4+ and 30% were Olig2+. ***, p < 0.0005; ****, p < 0.0001 versus CTL, 1-way ANOVA with Dunnett’s multiple comparison test.

**Figure 5.**
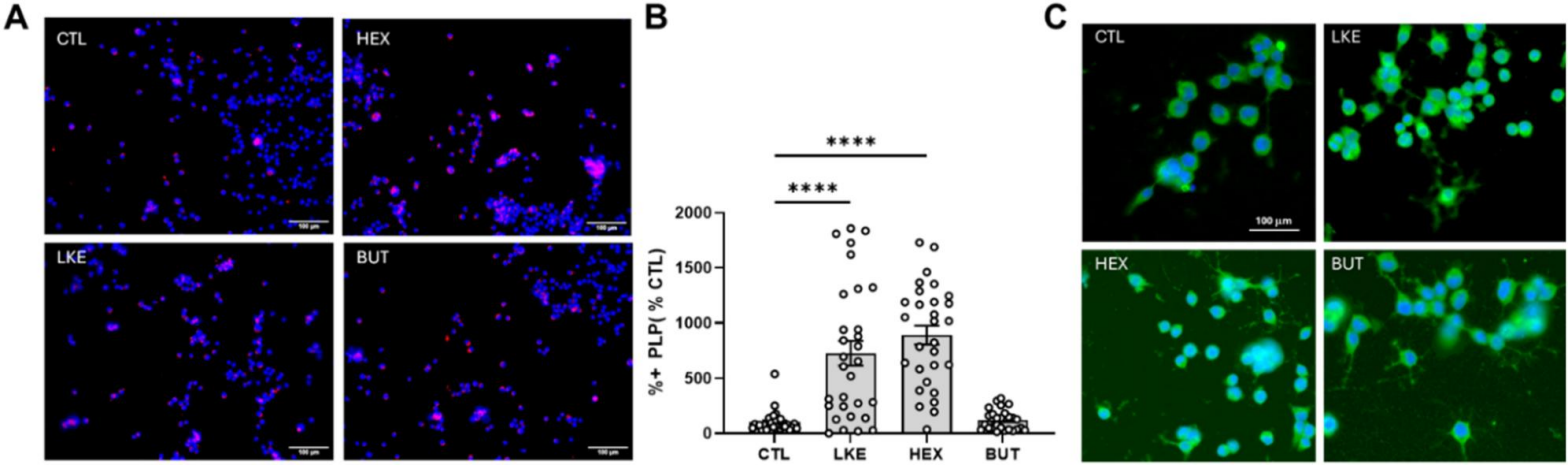
Incubation with LKE or HEX increases Oli-neu cell maturation. (**A**) Representative images of Oli-neu cells following incubation with 10 µM LKE, HEX, BUT, or nothing (CTL) for 72 hours, then stained for PLP (red) and counterstained with DAPI (blue). (**B)** Incubation with LKE or HEX significantly PLP staining. Data is mean ± se percent staining derived from each of 30 fields of view from 4 wells and normalized to staining in the CTL cells in which only 4.0% of the cells stained for PLP. ****, p < 0.0001 versus CTL, 1-way ANOVA with Dunnett’s multiple comparison test. (**C**) Representative images from a second study showing the presence of processes in LKE, HEX, and BUT cells. The brightness was increased on these to allow visualization of processes, but results in higher background staining.

**Figure 6.**
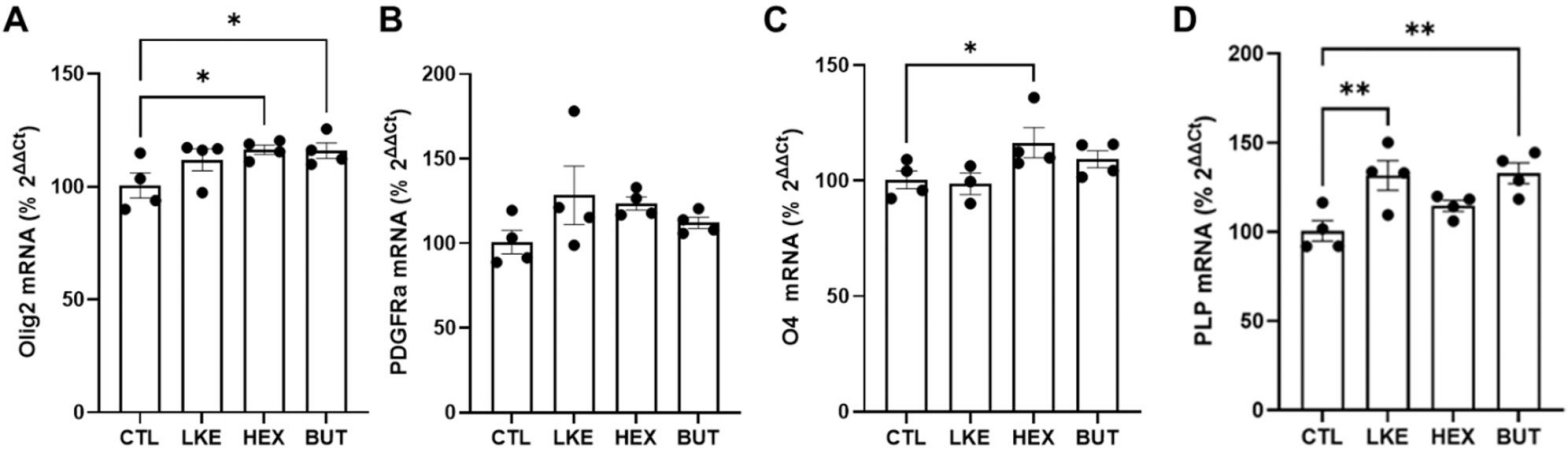
Effects of LKE, HEX, and BUT on Oli-neu relative mRNA expression. Oli-neu cells were incubated with 10 µM LKE, HEX, BUT, or nothing (CTL) for 24 hours, then mRNA isolated, converted to cDNA, and used to quantify mRNA levels of (**A**) Olig2, (**B**) PDGFRα, (**C**) O4, and (**D**) PLP relative to β-actin mRNA levels measured in the same sample. Data is mean ± se of n = 4 per group, normalized to values measured in CTL cells. Both Olig2 and PLP relative mRNA levels were increased by HEX and BUT; while O4 mRNA was increased by HEX. *, p < 0.05; **, p < 0.005 versus CTL, 1-way ANOVA with Dunnett’s multiple comparison test.

We compared the effects of BUT and HEX to LKE on Oli-neu cell proliferation and maturation (Figures 3–5). Initial studies done after 1 day of incubation did not reveal any changes in Ki67, PDGFRα, Olig2, or O4 staining. We therefore examined cells after 3 days incubation, at which time we observed a significant increase in Ki67 staining in the presence of LKE (135% of CTL) or HEX (181% of CTL), but not BUT (Figure 3B). We also observed a significant increase due to LKE (185% of CTL) or HEX (288% of CTL) in staining for PDGFRα, which is primarily expressed in immature OPCs (Figure 3C). The percentage of Ki67+:PDGFRα+ double-labeled cells averaged 50% in CTL cells and was significantly increased approximately 20% by LKE (Figure 3D). We observed increased staining for Olig2 following incubation with LKE (186% compared to CTL) or HEX (189% versus CTL) (Figure 4B). Staining for O4, present in immature OLGs as well as mature OLGs (Sommer & Schachner, 1982) was not altered following incubation with LKE or HEX, but was reduced by treatment with BUT (63% of CTL) (Figure 4C). In contrast, staining for PLP (Figure 5A), a marker of mature OLGs, was strongly increased (approximately 10-fold) by both LKE and HEX (Figure 5B). Incubation with LKE and HEX, as well as BUT also increased process outgrowth, an early morphological change occurring in OPCs (Thomason et al., 2020) (Figure 5C). Measurement of relative mRNA levels (Figure 6) showed that after 1 day incubation, the levels of both Olig2 and PLP mRNAs were increased by HEX and BUT, but not LKE. There was a modest, but non-significant increase in PDGFRα mRNA levels, and O4 mRNA levels were slightly increased by HEX.

**Figure 7.**
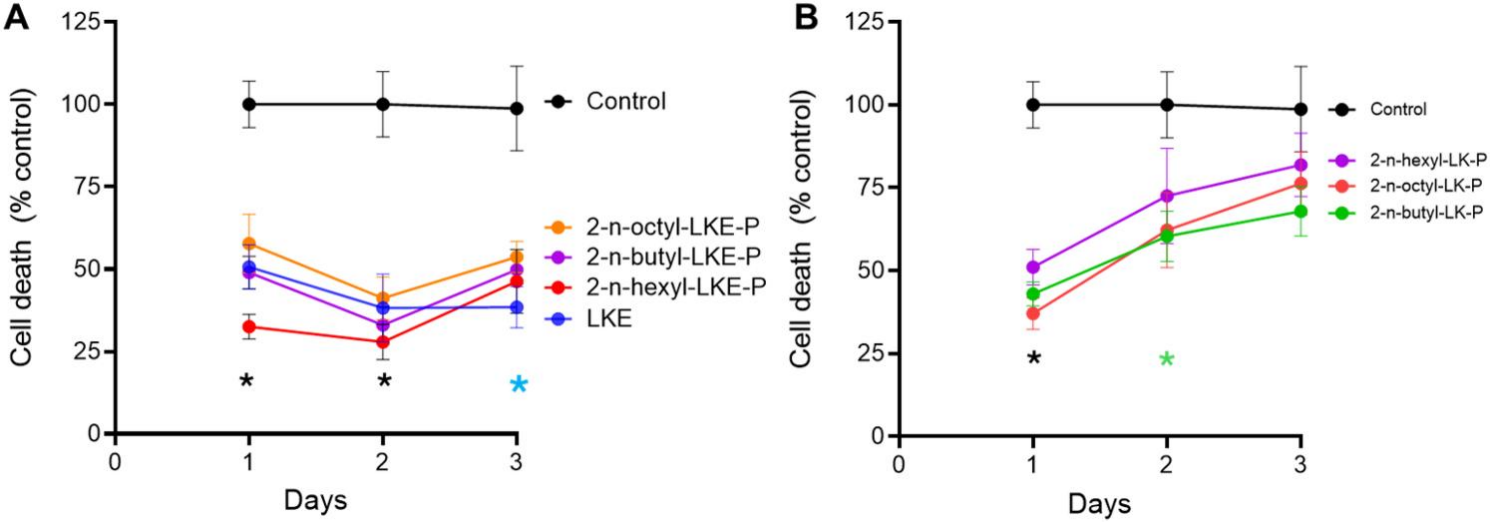
Relative toxicity of LK derivatives in primary OPCs. Primary mouse OPCs cells were incubated with 10 µM of (**A**) LKE and LKE-P derivatives and (**B**) LK-P derivatives, then cell death measured after indicated days by LDH release assay. For each day the data is presented as % cell death compared to untreated (CTL) cells, which was 13%, 16%, and 16% on days 1, 2, and 3 respectively. Data is mean ± se of n = 5 per group. On days 1 cell death was reduced by LKE, all 3 LKE-P and all 3 LK-P analogs. Cell death was reduced by LKE and the 3 LKE-P analogs on day 2, but only by LKE on day 3, and only by 2-*n*-butyl-LK-P on day 2. *, p < 0.05 versus CTL, 2-way repeated measures ANOVA with Dunnett’s multiple comparison test.

**Figure 8.**
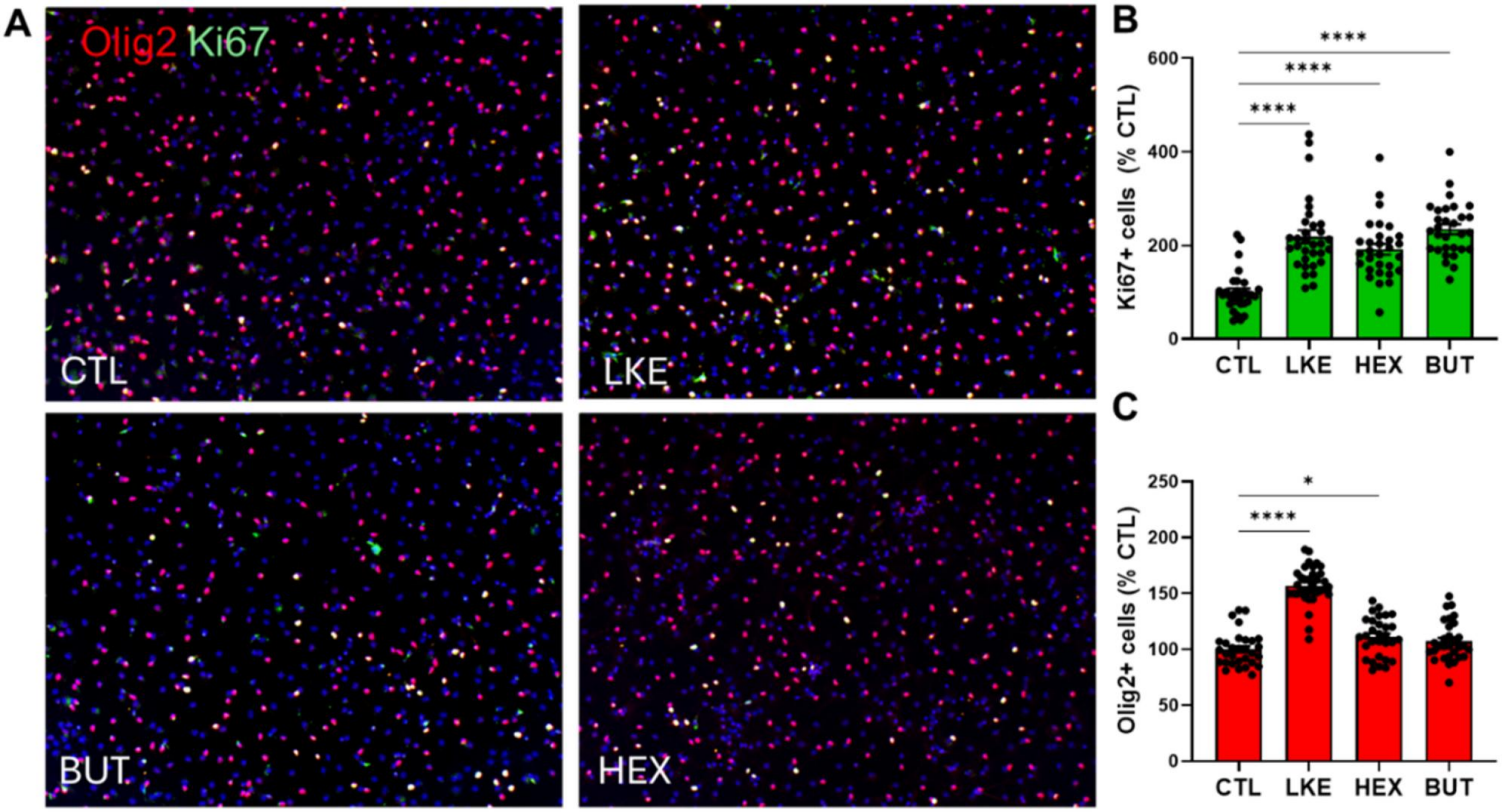
Effects of LKE, HEX, and BUT on OPC proliferation and total cell numbers. (**A**) Representative images of primary mouse OPCs after incubation with 25 µM LKE, HEX, BUT, or nothing (CTL) for 48 hours, then stained for Ki67 (green) or Olig2 (red), and counterstained with DAPI (blue). (**B**) Incubation with all analogs increased) Ki67 staining. (**C**) Only LKE significantly increased Olig2 staining. Data is mean ± se of the number of positively stained cells from 10 fields of view from 4 wells, normalized to number of cells in CTL (61.5 ± 4.9 per mm^2^ for Ki67; 296 ± 8.3 per mm^2^ for Olig2). *, p < 0.05; ****, p < 0.0001 versus CTL, 1-way ANOVA with Dunnett’s multiple comparison test.

**Figure 9.**
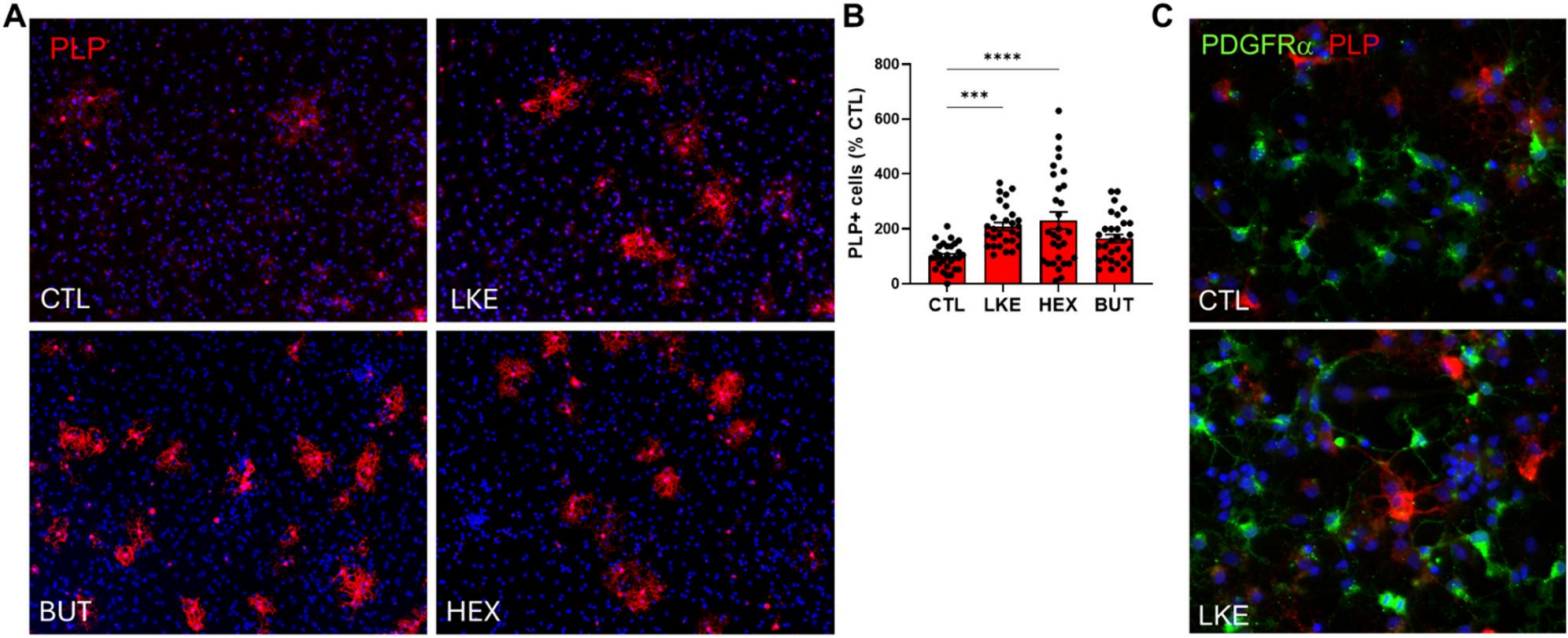
Effects of LKE, HEX, and BUT on OPC maturation. (**A**) Representative images of primary mouse OPCs after incubation with 25 µM LKE, HEX, BUT, or nothing (CTL) for 48 hours, then stained for PLP (red), and counterstained with DAPI (blue). (**B**) Incubation with LKE or HEX significantly increased the number of PLP stained cells. Data is mean ± se the number of positively stained cells derived from 10 fields of view from 4 wells, normalized to the number cells of PLP stained cells in CTL (9.5 ± 0.9 per mm^2^). ***, p < 0.001; ****, p < 0.0001 versus CTL, 1-way ANOVA with Dunnett’s multiple comparison test. (**C**) Co-staining of CTL and LKE-treated OPCs does not reveal co-express of PDGFRα (green) and PLP (red).

Since the properties and responses of Oli-neu cells can differ from those of primary OPCs, we examined the effects of LKE and analogs on primary mouse OPCs. As observed with Oli-neu cells, incubation with LKE or 3 LKE-P analogs significantly reduced spontaneous cell death occurring after 1 or 2 days, with the largest decreases observed with HEX and BUT (Figure 7A). In contrast, 3 of the LK-P analogs reduced cell death after 1 day; while only the 2-*n*-butyl-LK-P was able to decrease cell death after 2 days (Figure 7B). Based on these results, we tested the effects of LKE, BUT, and HEX on OPC proliferation and maturation (Figures 8 and 9). In initial studies we did not observe any significant effects when cells were incubated with 10 µM of the derivatives. However, compared to untreated (CTL) cells, each of these analogs when used at 25 µM significantly increased Ki67 staining (Figure 8B). Staining for Olig2 (Figure 8C) showed that LKE significantly increased the total number of OLGs, with a modest but significant increase observed with HEX. As found for Oli-neu cells, incubation with either LKE or HEX significantly increased the number of cells staining for PLP (Figure 9A and B).

Since LKE and HEX increased staining for both PDGFRα and PLP, we tested if that occurred in distinct cell populations or if there was any indication of co-expression. We used primary OPCs since results in these better reflect the physiological situation. Double-labeling shows that in both CTL OPCs and LKE-treated OPCs there was no evidence of co-expression (Figure 9C), indicating that LKE and HEX increased expression in different cells.

## Discussion

The current results demonstrate that in addition to LKE, other LK analogs have the potential to induce OPC proliferation and maturation. Screening for possible toxic effects of these analogs in Oli-neu cells revealed a transient increase in endogenous cell death after 1 day incubation with HEX or BUT; followed by significant reductions in cell death after 2 or 3 days of incubation. Since in these studies, the LK derivatives were only added once at the beginning of the study, early toxicity may have been due to exposure to the initial high dose (10 µM) which was reduced over the following days due to chemical or enzymatic breakdown. While the causes for background cell death in Oli-neu are not known, the data indicate that LKE and LKE-P derivatives show little or no toxicity and can reduce the spontaneous cell death. In primary mouse OPCs, none of the LK analogs increased spontaneous cell death which was approximately 15%. All analogs reduced cell death after 1 day; the LKE-P derivatives reduced death after 2 days, while only LKE reduced death after 3 days and 2-*n*-butyl-LK-P after 2 days. These findings again indicate that LKE, HEX, and BUT are the best candidates for further studies in OPCs.

Mouse Oli-neu cells have been used extensively as a model for OPCs; and in many studies the responses of Oli-neu cells to various stimuli reflected those that occur in primary OPCs. Oli-neu cells can be differentiated into mature OLGs by dbcAMP, express canonical OLG markers, and the mature cells interact and ensheath axons demonstrating physiological normal OLG functions (Jung et al., 1995). Similarly, overexpression of opalin, which is strongly expressed during early OPC differentiation, induced expression in Oli-neu cells of MAG, CNP, and CGT (de Faria et al., 2019). Oli-neu cells also express several calcium channels (Cav1.2) that are expressed in OPCs and OLGs. (Enders et al., 2023). Our results show that Oli-neu proliferation, assessed by Ki67 expression, was increased following incubation with HEX and modestly increased upon incubation with LKE. Similar to Ki67 expression, PDGFRα, which is strongly expressed in OPCs and whose expression is reduced with maturation, was increased in Oli-neu cells by HEX, with a smaller increase due to LKE. These results indicate that some LK derivatives increase Oli-neu cell proliferation leading to an increase of PDGFRα staining. Since Olig2 is expressed in all cells of the OLG lineage, the increase in cell numbers is expected to be associated with a concomitant increase in Olig2 staining. As found for Oli-neu cells, incubation with LKE, HEX, or BUT increased Ki67 staining in primary mouse OPCs (Figure 8B). Although we did not carry out staining for PDGFRα, staining for Olig2 (Figure 8C) showed an increase in the total number of OLGs due to LKE, and a modest increase due to HEX. This suggests that, as in Oli-neu cells, LKE and HEX increase proliferation of pre-existing immature cells that do not express Olig2. However, it is possible that reductions in cell death contribute to the observed increases.

We previously showed that following cuprizone induced demyelination, the extent of subsequent remyelination was increased in the presence of LKE (Dupree et al., 2022). In that study, after 2 weeks of remyelination immunostaining of sections from the Corpus Callosum showed an increased number of mature OLGs (CC1+/Olig2+ cells) in samples from mice provided LKE during the remyelination period as compared to samples from mice kept on control chow, although there was no increase in the percentage of proliferating OPCs (Ki67+/Olig2+). Since those analyses were done at a single time point (after 2 weeks of remyelination), our in vitro findings that HEX, as well as LKE to a lesser extent increase OPC proliferation suggests that in vivo, LKE may have increased proliferation at an earlier time during remyelination.

Neither LKE nor HEX affected the number of cells that stained for the cell surface marker O4, although incubation with BUT slightly decreased staining. In contrast, incubation with LKE or HEX led to an increase in the number of PLP positively stained cells. O4 expression begins at the pro-oligodendroblast stage, and is maintained in immature and mature OLGs (Lin et al., 2006); therefore induction of maturation from early, immature OLGs to mature OLGs would not be reflected by an increase in O4 expression. In contrast, PLP is mainly expressed in mature OLGs; increased PLP staining therefore suggests maturation of O4 positive/PLP negative cells to O4 positive/PLP positive stained cells. We observed increases in PLP as well as PDGFRα, an unexpected finding as maturation to PLP expressing cells would be expected to reduce the number of PDGFRα+ stained cells. There are several possible explanations for this, the most parsimonious that Oli-neu cultures consist of a heterogenous population of cells at different stages of maturation. LKE and HEX could induce PDGFRα expression in pre-existing or newly-generated PDGRFα-negative cells, while at the same time induce PLP expression in more mature cells. It was also reported that PLP is expressed in early progenitor cells (LeVine et al., 1990), is downregulated as cells migrate, and then re-expressed during OPC differentiation into mature OLGs (Harlow et al., 2014). In addition, it is possible that the rabbit polyclonal antibody we used to detect PLP also detects DM-20, an alternatively spliced form of PLP expressed during early stages of OPC maturation (Griffiths et al., 1995; LeVine et al., 1990). The function of PLP in OPCs is not clear, but may play a role in migration and process extension (Yang & Skoff, 1997). This leads to an interesting possibility that LKE induces PLP and/or DM-20 expression in immature OPCs. An increase in PLP expressing cells due to LKE and HEX was also observed in primary mouse OPCs (Figure 9B); however, the magnitude of that increase (approximately 2-fold) was less that that in Oli-neu cells (between 6- to 8-fold). While we have not yet quantified effects of LKE or HEX on OPC PDGFRα expression, our results of double-labeling for PDGFRα and PLP in OPCs supports that the increased expression occurs in different cell populations (Figure 9C). Overall, our findings indicate that LKE and derivatives can induce maturation of OPCs and highlight differences in Oli-neu cells as compared to primary cells.

We observed that all 3 LK analogs induced process formation and elongation in Oli-neu cells. LKE and HEX also increased the percentage of PLP-expressing cells, suggesting a correlation with process formation. However, we did not observe any significant increase of PLP expressing cells due to BUT. This could reflect the fact that process outgrowth in OPCs precedes myelin gene expression, and that morphological changes can be regulated distinctly from the processes of myelin gene transcription (Thomason et al., 2020). Mechanistically, this could involve effects of the LK derivatives on the ability of CRMP2 to regulate actin polymerization (Arimura et al., 2005; Takizawa et al., 2025) or semaphorin signaling which can induce OPC migration (Piaton et al., 2011; Stratton et al., 2020; Syed et al., 2017). Overall, the results suggest that BUT may be acting to induce OPC maturation at a slower rate than either LKE or HEX; examination of cells at later times will be able to address this possibility.

Quantitation of relative mRNA levels showed that incubation with HEX for 1-day increased Olig2, PLP, and O4 mRNA levels in Oli-neu cells. Modest increases were observed for PDGFRα however those were not significantly greater than control values. The absence of an increase of O4 staining suggests increased O4 mRNA may not be efficiently translated, or that increases in O4 protein expression may occur at later times. Together, the data suggests that compared to LKE, treatment with 2-*n*-hexyl-LKE(P) leads to equivalent or possibly greater induction of proliferation as well as maturation of OPCs. Whether longer incubation leads to maturation of fully mature OLGs remains to be determined. In view of its stronger hydrophobicity and greater potential to cross the blood brain barrier, this suggests HEX may be a preferred analog for testing in animal models of MS.

The LK analogues 2-*n*-butyl-LKE-P and 2-*n*-Hexyl-LKE-P were originally generated along with a number of other derivatives (Shen et al., 2018); however, studies examining their properties are limited. Several analogs including HEX were recently shown to modulate autophagy in a motor neuron hybrid cell line (Gonzalez Porras et al., 2023). When tested alone, none of the analogs influenced autophagy flux, or the ratio of autophagy proteins LC3II: LC3I. However, when loaded into nanoparticles autophagy was greatly enhanced compared to derivatives or nanoparticles alone. While direct comparison of the 3 analogs to each other was not done, the effects of HEX on indices of autophagy were as great or greater than that observed with the other 2 analogs. It was speculated that in this system, intracellular delivery of the LK analogs was necessary to observe effects. Our findings that LK analogues alone are able to influence Oli-neu cells suggests that encapsulation into nanoparticles could further increase their proliferative and maturation effects.

## Data Availability

The authors confirm that the data supporting the findings of this study are available within the article and any raw data not presented in the article is also available from the corresponding author (DLF) upon request.
